# Supplementary figures and images for: Nrf2 deficiency causes hepatocyte dedifferentiation and reduced albumin production in an experimental extrahepatic cholestasis model
Source: PLoS One. 2022 Jun 13;17(6):e0269383. doi: 10.1371/journal.pone.0269383 (PMC9191739; doi:10.1371/journal.pone.0269383)

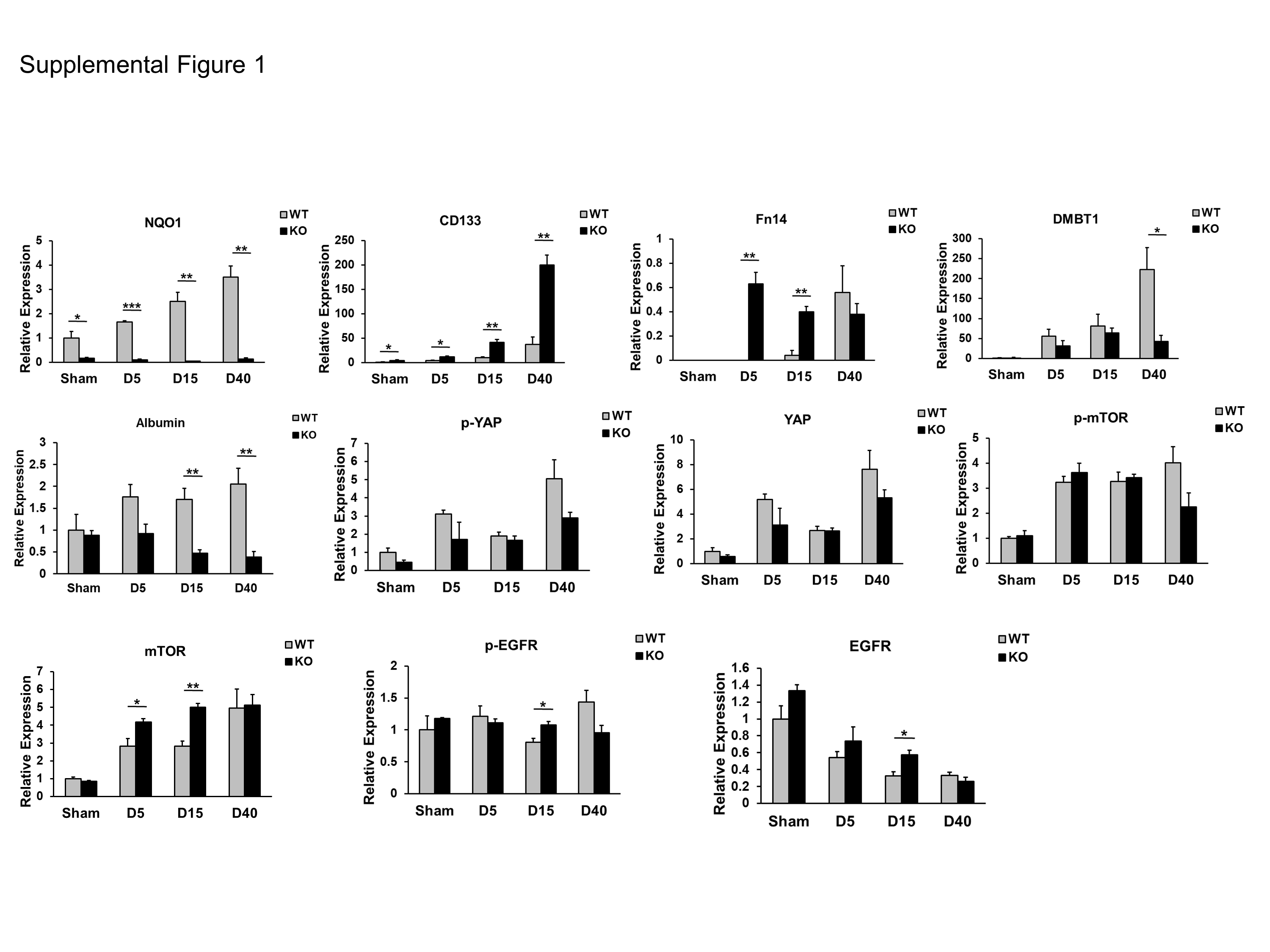

Supplement: S1 Fig — Data are presented as means ± SD normalized with the loading controls and relative to the sham controls (day 40 after surgery) (n = 3). *P < 0.05; **P < 0.01; ***P < 0.001. (TIF) [file pone.0269383.s001.tif]
