# Supplementary material for: Nrf2 deficiency causes hepatocyte dedifferentiation and reduced albumin production in an experimental extrahepatic cholestasis model
Source: PLoS One. 2022 Jun 13;17(6):e0269383. doi: 10.1371/journal.pone.0269383 (PMC9191739; doi:10.1371/journal.pone.0269383)
Supplement: S1 Raw images — (PDF) [file pone.0269383.s002.pdf]

Fig 6B Original Western Images with Molecular Weight Markers

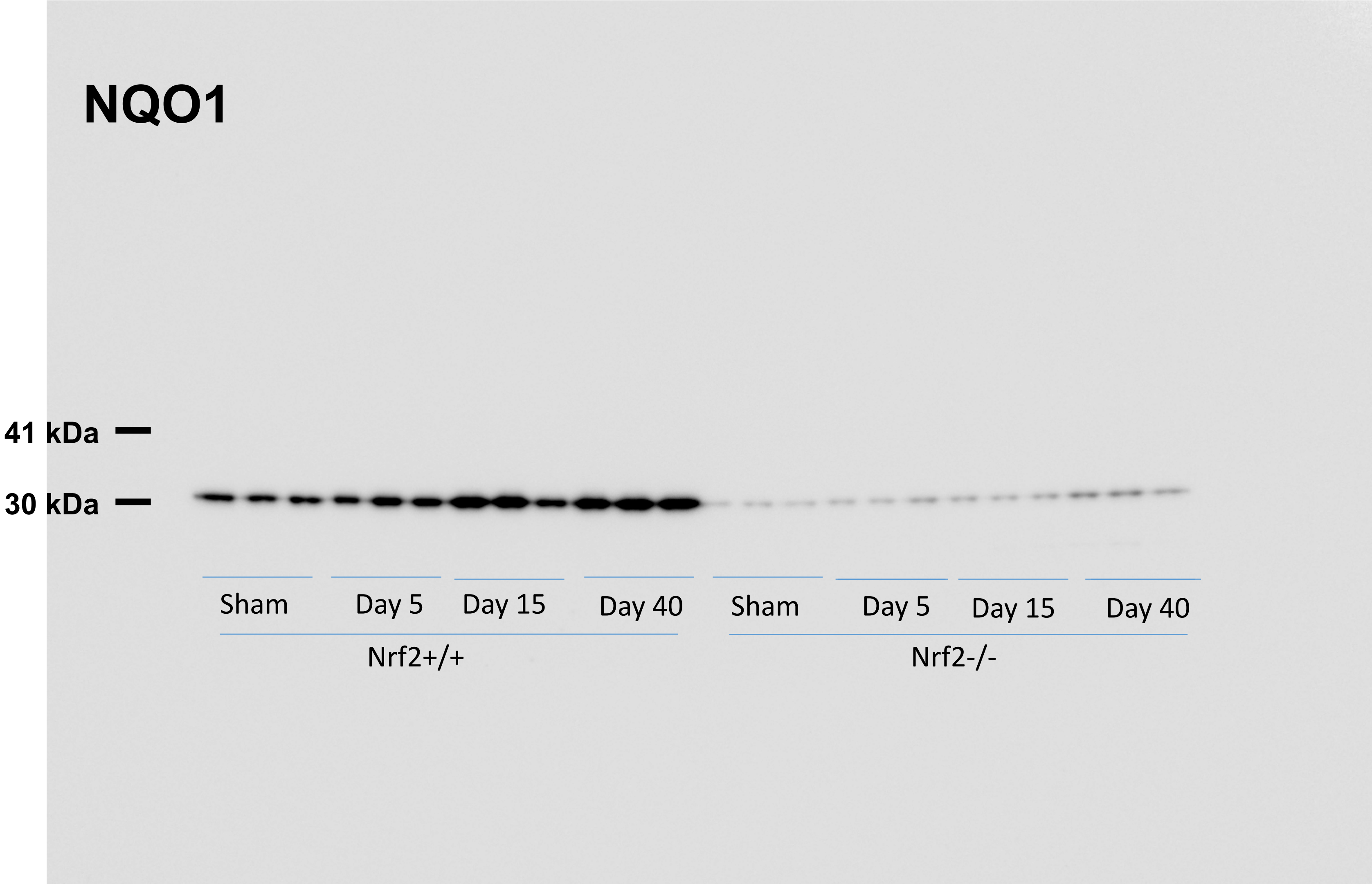
NQO1
41 kDa
30 kDa
Sham
Day 5
Day 15
Day 40
Sham
Day 5
Day 15
Day 40
Nrf2+/+
Nrf2-/-

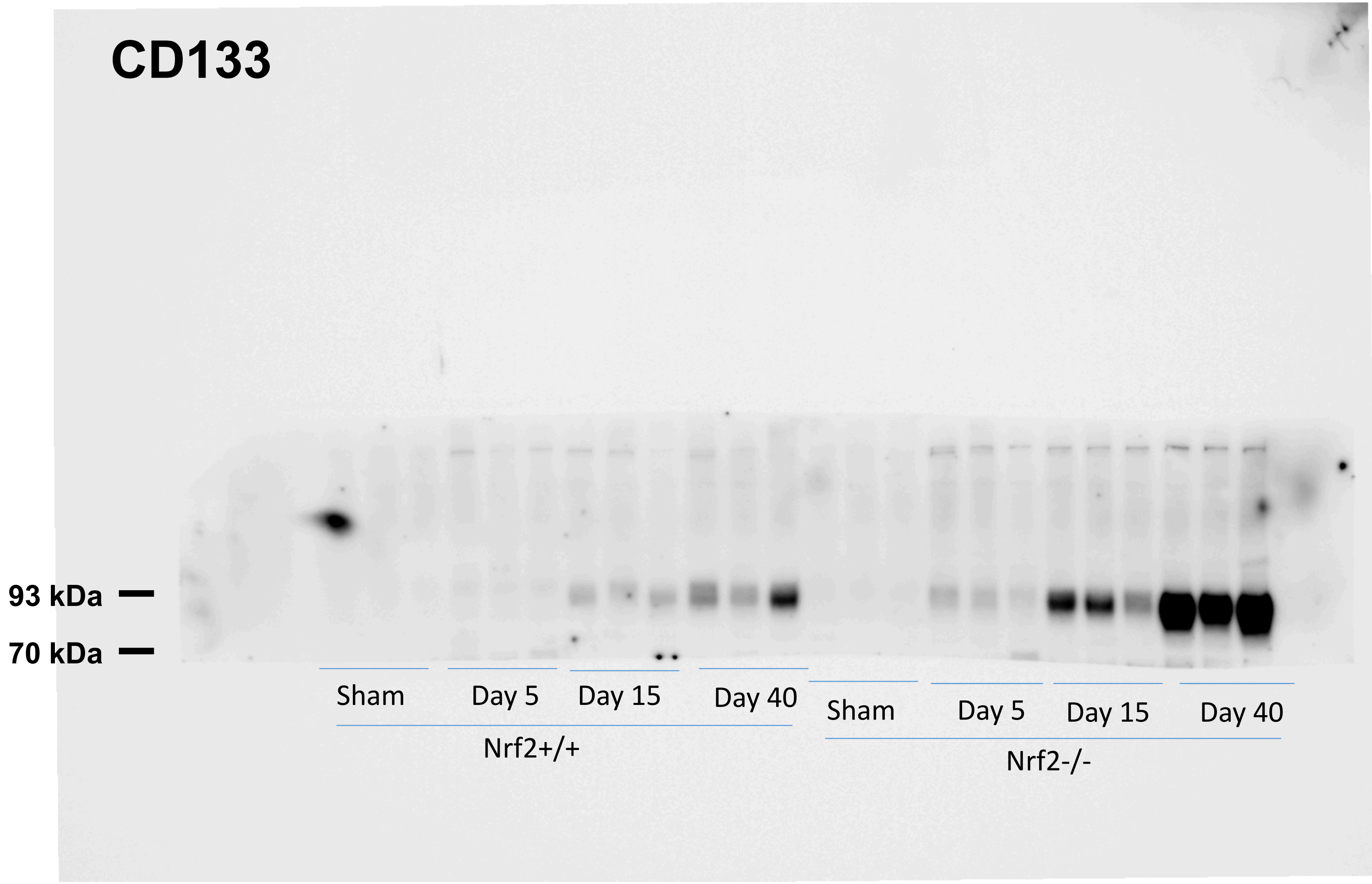

CD133
93 kDa
70 kDa
Sham
Day 5
Day 15
Day 40
Nrf2+/+
Sham
Day 5
Day 15
Day 40
Nrf2-/-

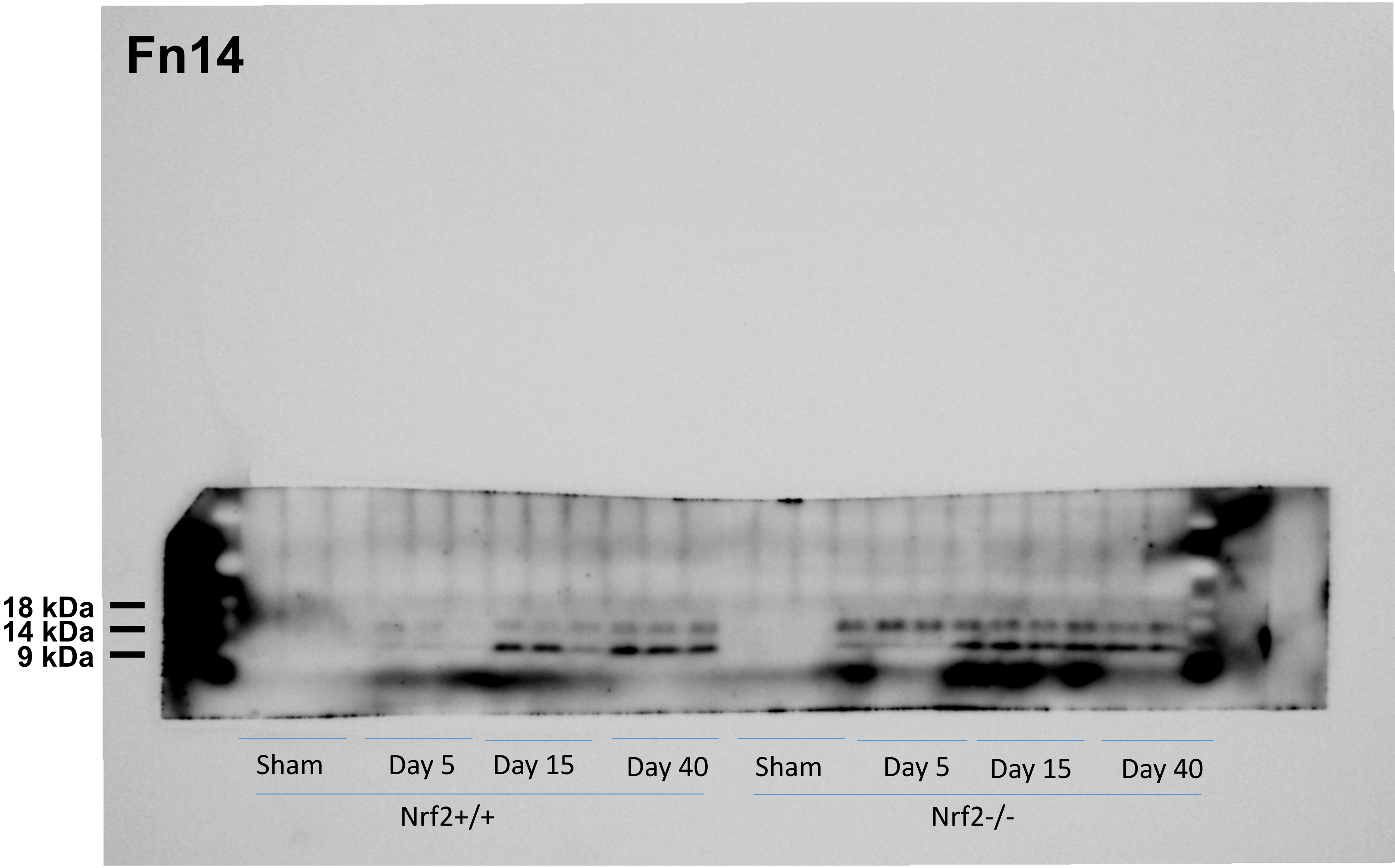
Fn14
18 kDa
14 kDa
9 kDa
Sham
Day 5
Day 15
Day 40
Sham
Day 5
Day 15
Day 40
Nrf2+/+
Nrf2-/-

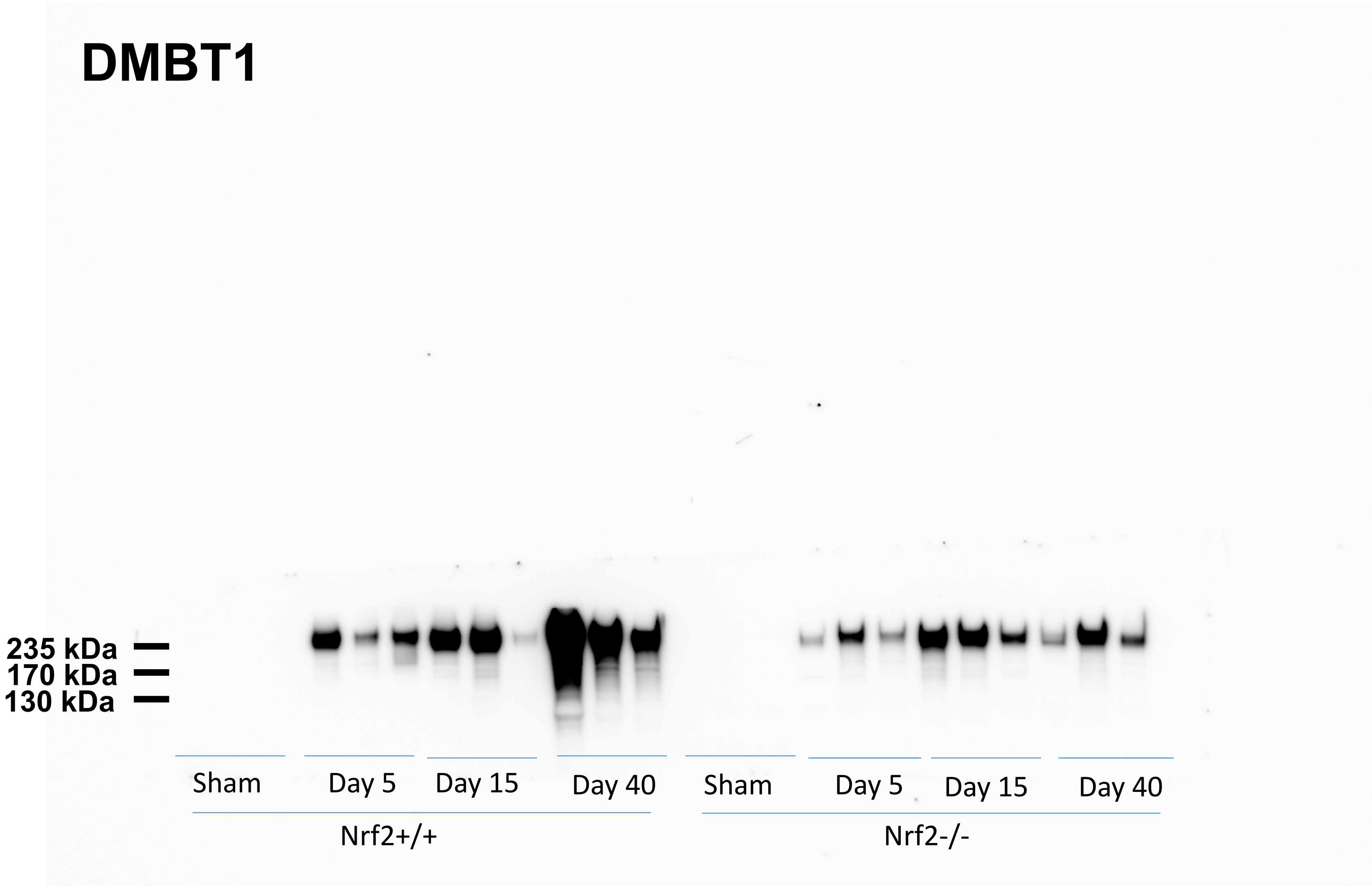
DMBT1
235 kDa
170 kDa
130 kDa
Sham
Day 5
Day 15
Day 40
Sham
Day 5
Day 15
Day 40
Nrf2+/+
Nrf2-/-

Albumin

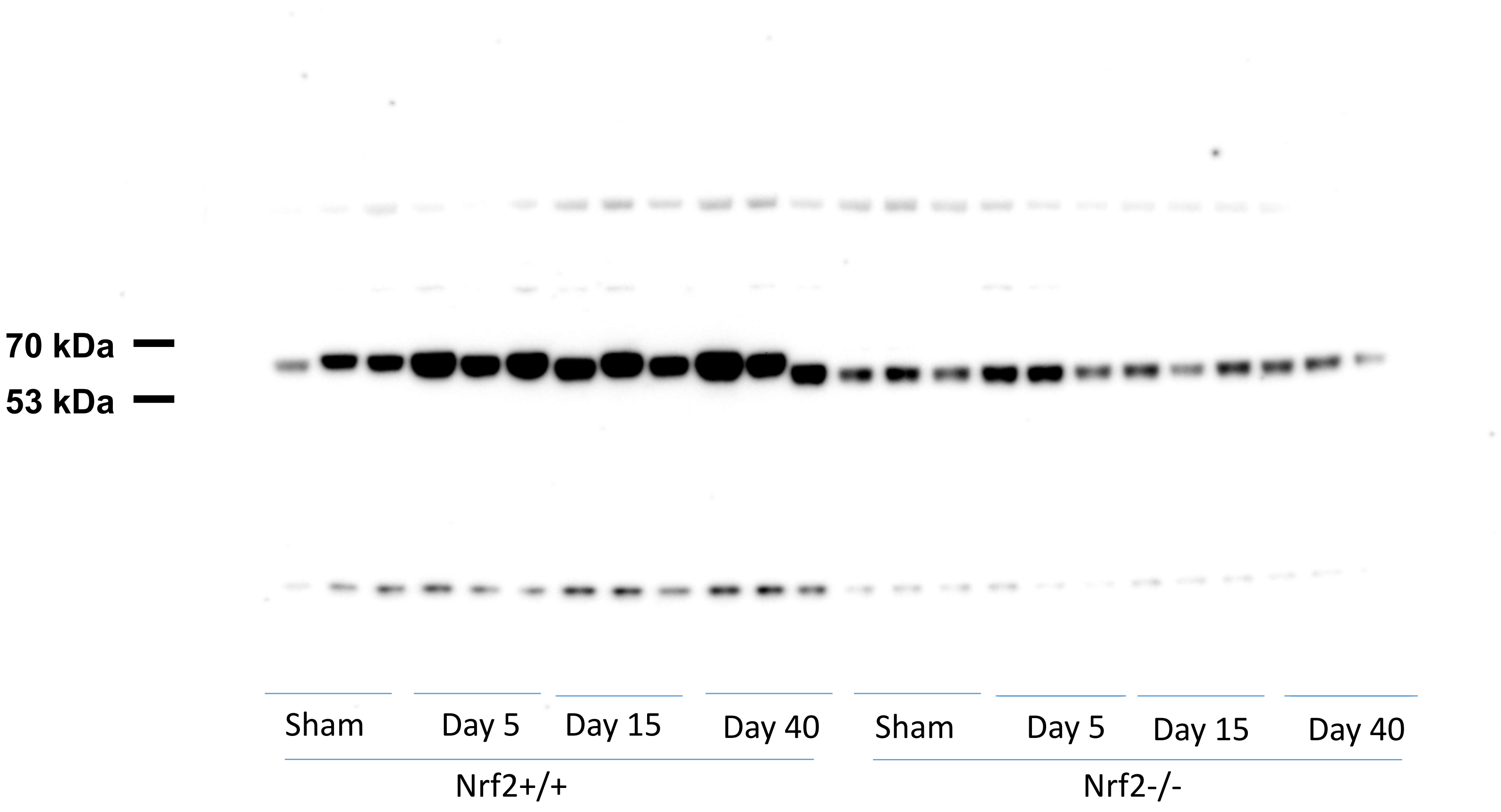

70 kDa
53 kDa
Sham
Day 5
Day 15
Day 40
Sham
Day 5
Day 15
Day 40
Nrf2+/+
Nrf2-/-

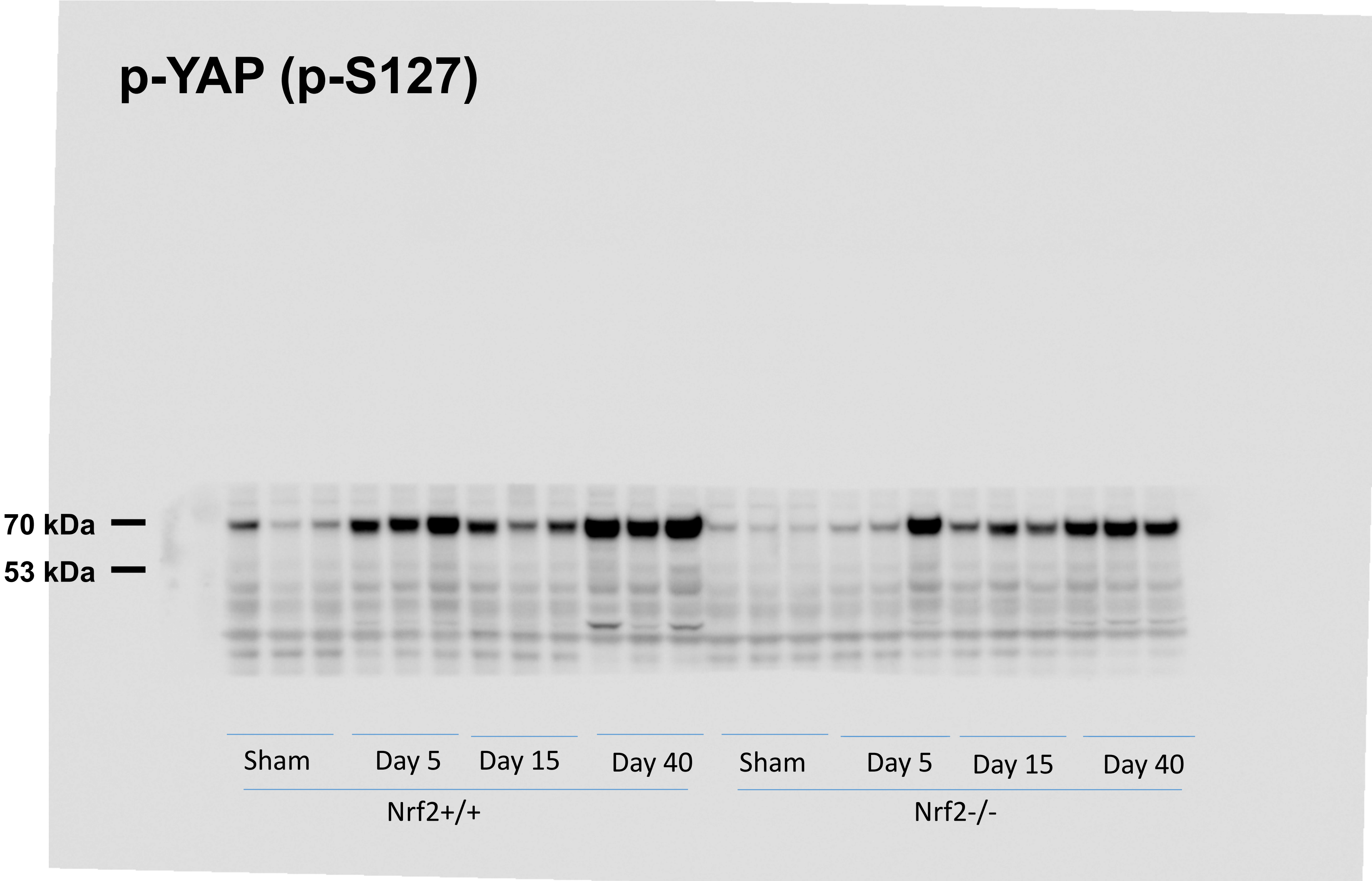
p-YAP (p-S127)
70 kDa
53 kDa
Sham
Day 5
Day 15
Day 40
Sham
Day 5
Day 15
Day 40
Nrf2+/+
Nrf2-/-

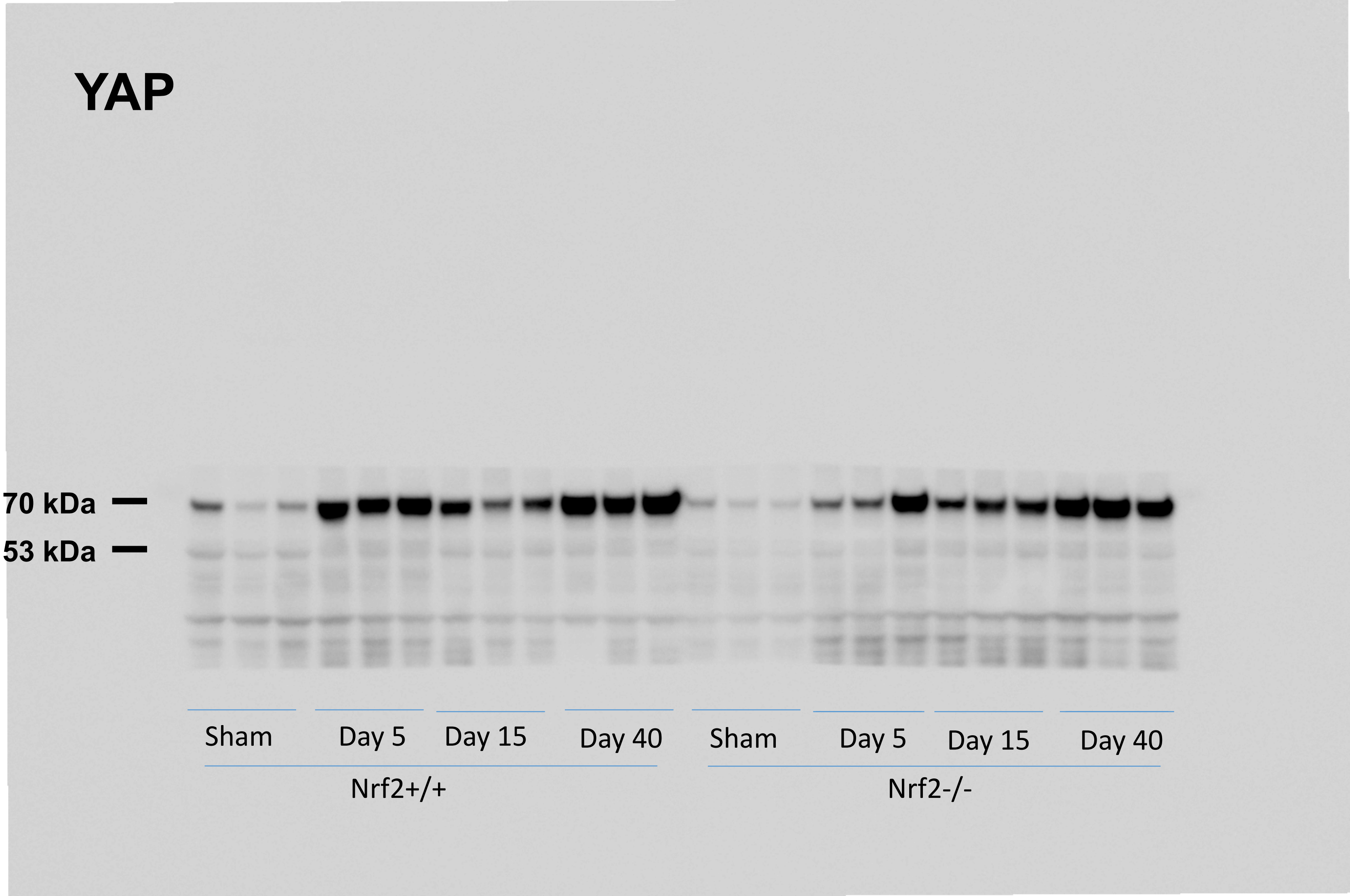
YAP
70 kDa
53 kDa
Sham
Day 5
Day 15
Day 40
Sham
Day 5
Day 15
Day 40
Nrf2+/+
Nrf2-/-

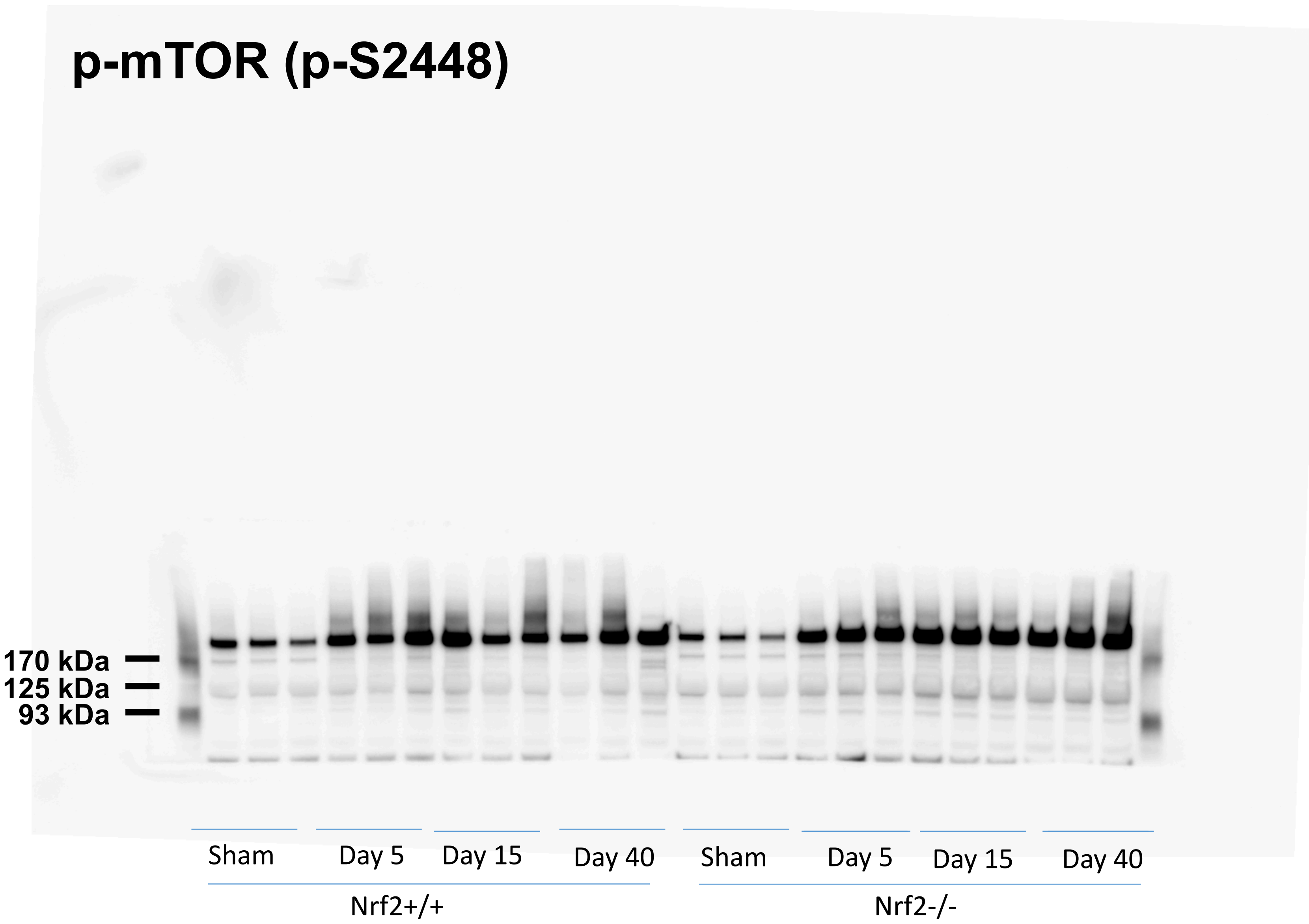
p-mTOR (p-S2448)
170 kDa
125 kDa
93 kDa
Sham
Day 5
Day 15
Day 40
Sham
Day 5
Day 15
Day 40
Nrf2+/+
Nrf2-/-

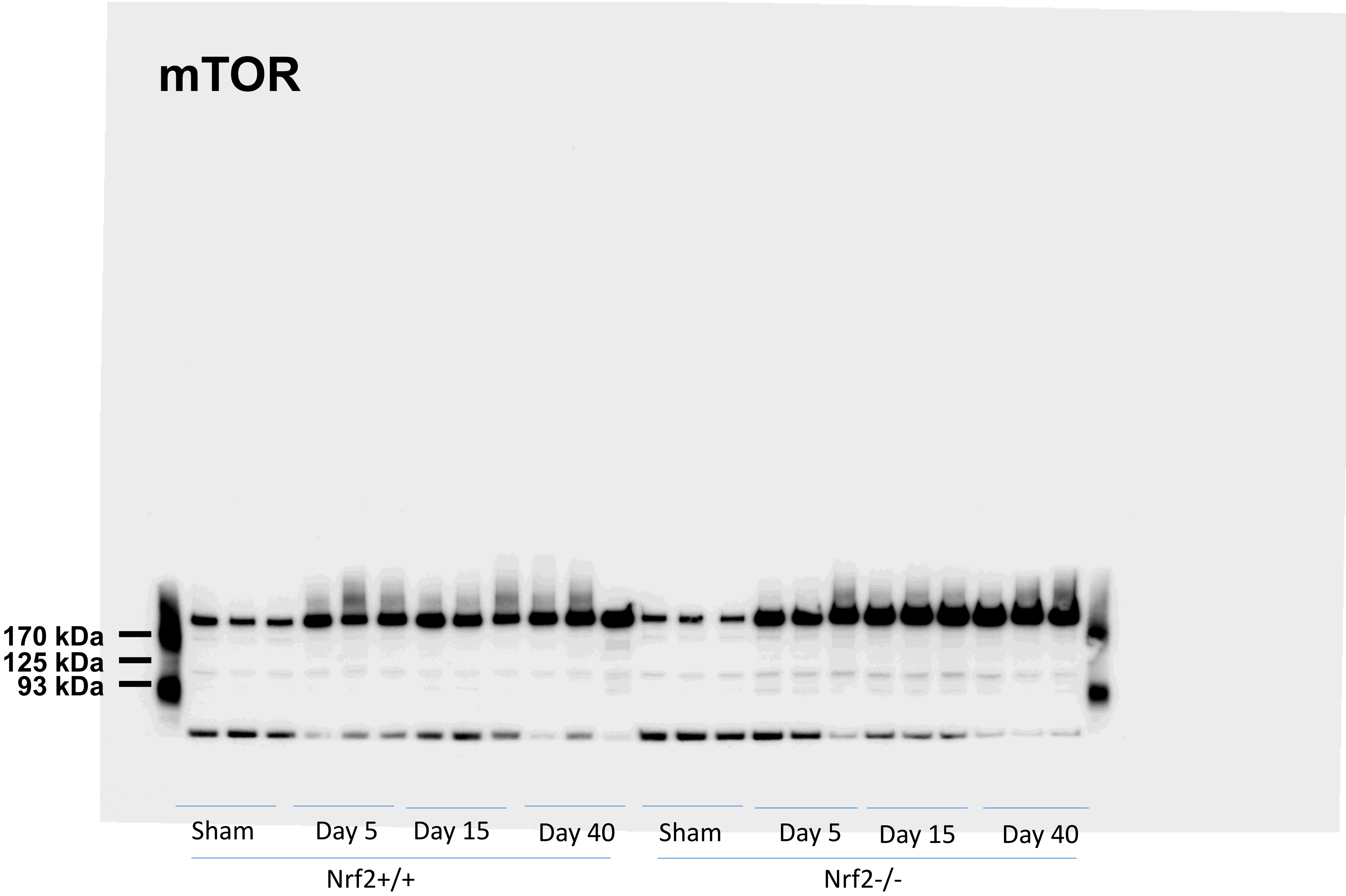
mTOR
170 kDa
125 kDa
93 kDa
Sham
Day 5
Day 15
Day 40
Sham
Day 5
Day 15
Day 40
Nrf2+/+
Nrf2-/-

p-EGFR (p-Y1086)

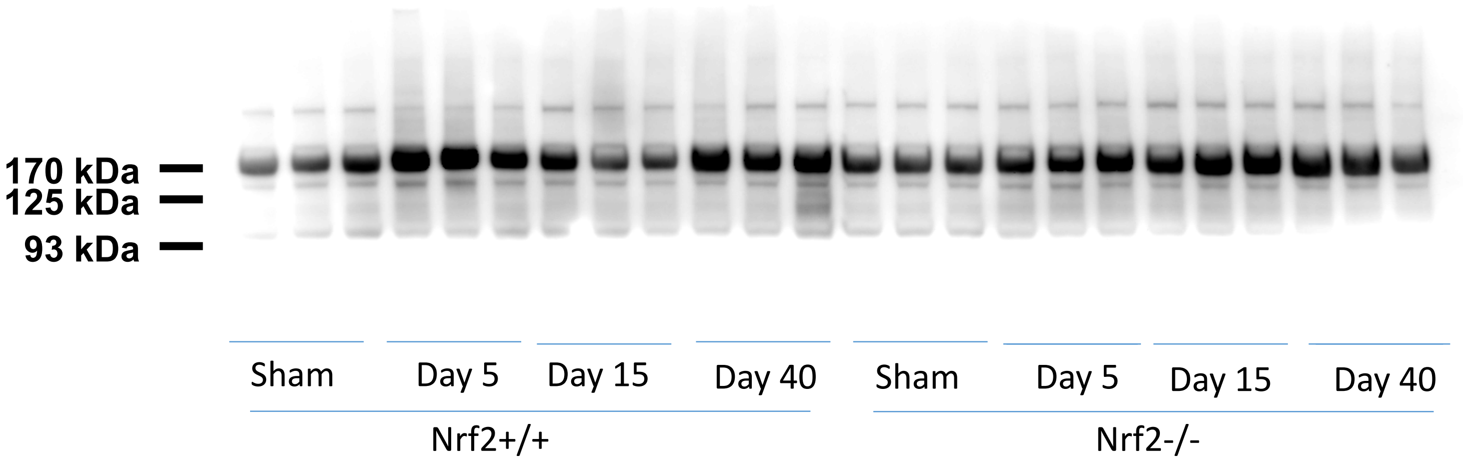

EGFR

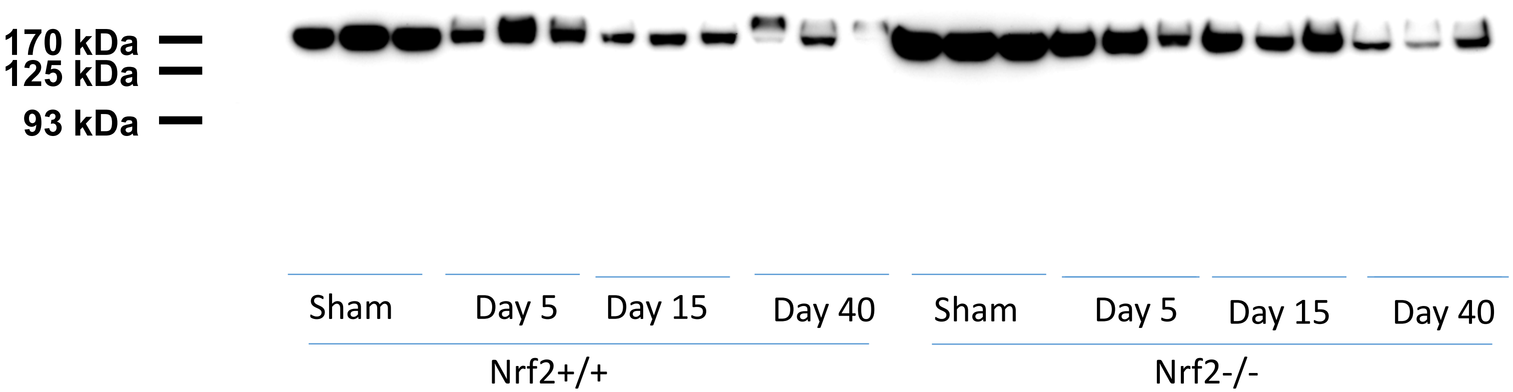
170 kDa
125 kDa
93 kDa
Sham
Day 5
Day 15
Day 40
Sham
Day 5
Day 15
Day 40
Nrf2+/+
Nrf2-/-

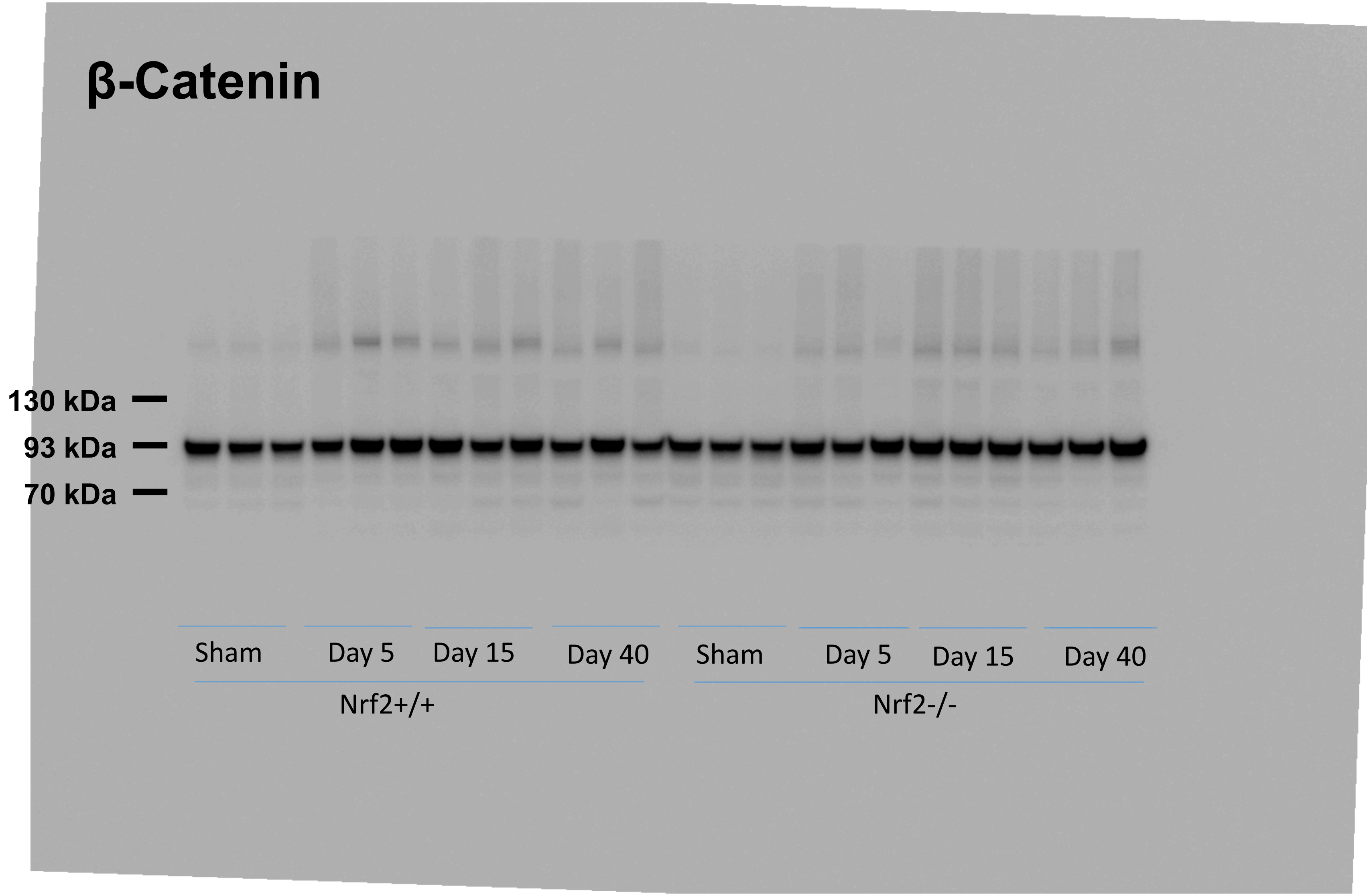
β-Catenin
130 kDa
93 kDa
70 kDa
Sham
Day 5
Day 15
Day 40
Sham
Day 5
Day 15
Day 40
Nrf2+/+
Nrf2-/-
